# Supplementary figures and images for: Salivary microbial changes during the first 6 months of orthodontic treatment
Source: PeerJ. 2020 Dec 1;8:e10446. doi: 10.7717/peerj.10446 (PMC7718796; doi:10.7717/peerj.10446)

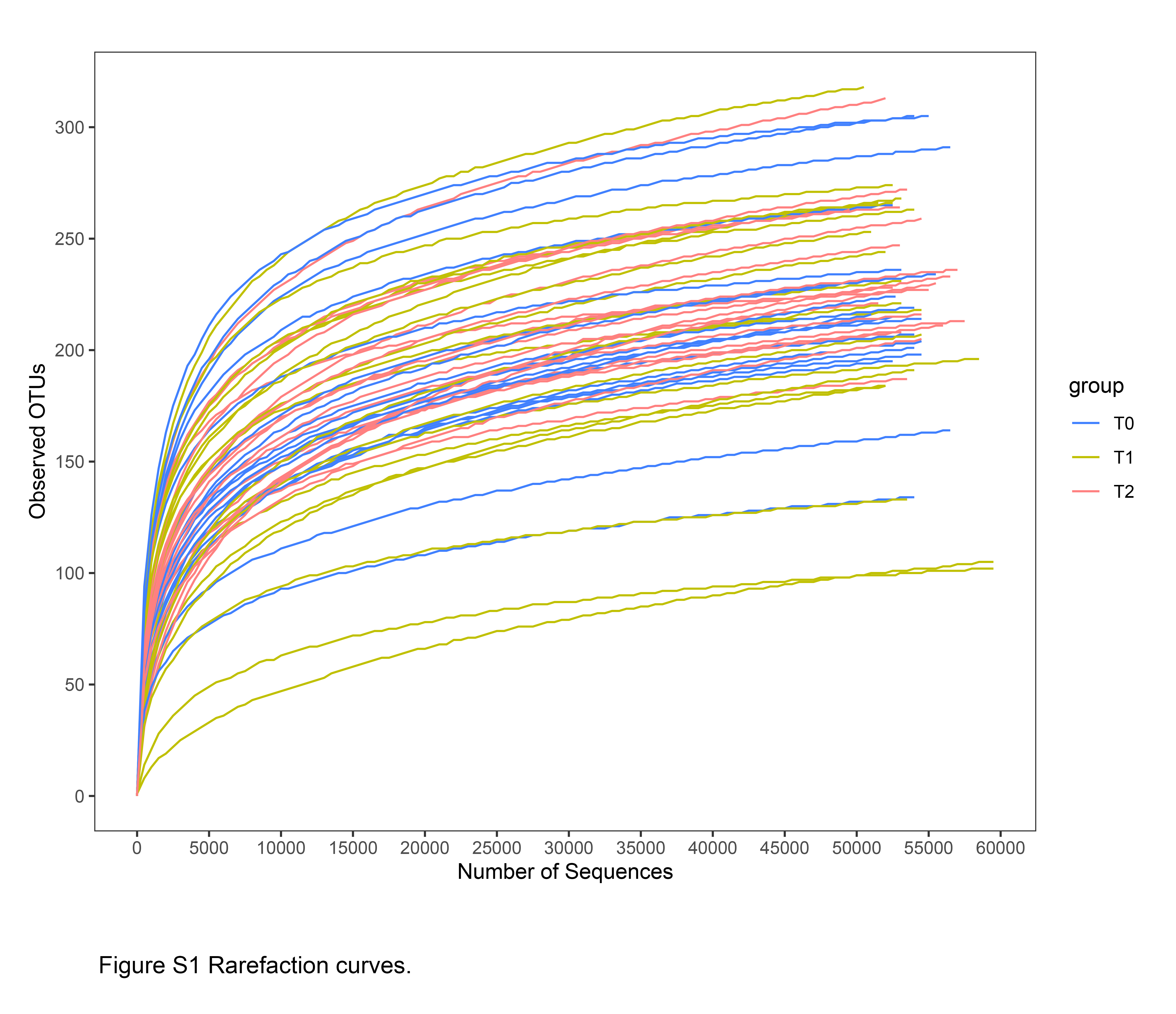

Supplement: Supplemental Information 1 [file peerj-08-10446-s001.jpg]

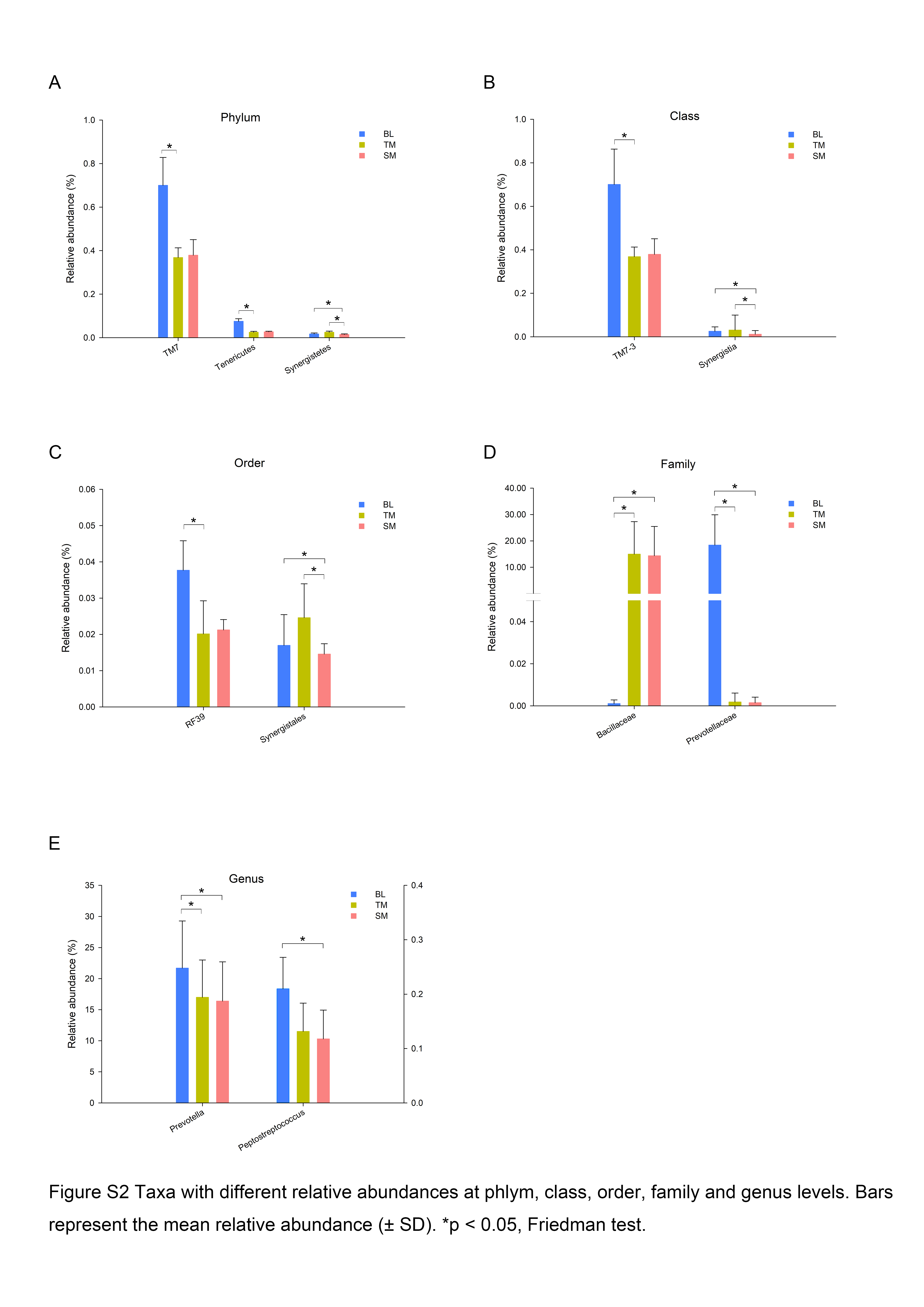

Supplement: Supplemental Information 2 — Bars represent the mean relative abundance (±SD). ∗p < 0.05, Friedman test. [file peerj-08-10446-s002.jpg]
