# Supplementary material for: Salivary microbial changes during the first 6 months of orthodontic treatment
Source: PeerJ. 2020 Dec 1;8:e10446. doi: 10.7717/peerj.10446 (PMC7718796; doi:10.7717/peerj.10446)
Supplement: Supplemental Information 3 [file peerj-08-10446-s003.pdf]

Table S1-1 Relative abundances of OTUs

	OTU ID and taxa	Relative abundance (%)
T0 unique (6)	OTU046: S24-7	0.0025
	OTU146: Alphaproteobacteria	0.0014
	OTU158: Peptostreptococcaceae	0.0004
	OTU164: S24-7	0.0004
	OTU187: Tissierellaceae	0.0004
	OTU188: Moryella	0.0005
T1 unique (0)	none	0
T2 unique (1)	OTU011: Coriobacteriaceae	0.0011
T0 & T1 (8)	OTU121: Treponema	0.0012
	OTU127: TM7-3	0.0007
	OTU152: Lactobacillus	0.0005
	OTU184: SHD-231	0.0006
	OTU217: Oribacterium	0.0003
	OTU247: Lactococcus	0.0003
	OTU291: Acidovorax	0.0003
	OTU292: Mogibacteriaceae	0.0004
T1 &T2 (4)	OTU137: Moraxella	0.0009
	OTU179: Alloscardovia	0.0003
	OTU283: Prevotella	0.0004
	OTU338: Veillonellaceae	0.0004
T0 & T2 (6)	OTU061: Leptotrichia	0.0035
	OTU125: Acholeplasma	0.0014
	OTU135: Corynebacterium	0.0014
	OTU168: Treponema	0.0012
	OTU195: Syntrophomonas	0.0004
	OTU204: Unclassified	0.0005
