# Supplementary material for: Salivary microbial changes during the first 6 months of orthodontic treatment
Source: PeerJ. 2020 Dec 1;8:e10446. doi: 10.7717/peerj.10446 (PMC7718796; doi:10.7717/peerj.10446)
Supplement: Supplemental Information 4 [file peerj-08-10446-s004.pdf]

Table S1-2 Information of core microbiome

OTU ID and taxa	Relative abundance (%)
OTU278: Neisseria	9.4677
OTU368: Prevotella	9.3423
OTU192: Streptococcus	6.8236
OTU238: Haemophilus	5.4626
OTU321: Veillonella	4.7226
OTU389: Rothia	3.4661
OTU013: Fusobacterium	2.8501
OTU342: Veillonella	1.6545
OTU214: Prevotella	0.7239
OTU104: Prevotella	0.7057
OTU249: Granulicatella	0.6802
OTU272: Haemophilus	0.6186
OTU310: Actinomyces	0.5346
OTU038: Rothia	0.4988
OTU170: Campylobacter	0.4595
OTU200: Gemellaceae	0.3356
OTU113: Lautropia	0.3352
OTU293: Atopobium	0.2225
OTU408: Selenomonas	0.2202
OTU154: Lachnoanaerobaculum	0.1876
OTU351: Oribacterium	0.1687
OTU253: Peptostreptococcus	0.1530
OTU329: Mogibacteriaceae	0.1480
OTU177: Leptotrichia	0.1228
OTU385: Catonella	0.0721
OTU287: Fusobacterium	0.0654
