# Supplementary material for: Salivary microbial changes during the first 6 months of orthodontic treatment
Source: PeerJ. 2020 Dec 1;8:e10446. doi: 10.7717/peerj.10446 (PMC7718796; doi:10.7717/peerj.10446)
Supplement: Supplemental Information 5 [file peerj-08-10446-s005.pdf]

Table S2-1 Information of hub nodes (T0)

OTU ID and taxa	Relative abundance (%)
OTU028: Prevotella	0.0286
OTU030: Actinomyces	0.0099
OTU041: Capnocytophaga	0.0080
OTU047: Capnocytophaga	0.0812
OTU053: Prevotella	0.0153
OTU057: Leptotrichia	0.0203
OTU069: Peptostreptococaceae	0.0241
OTU074: Aggregatibacter	0.1406
OTU120: Leptotrichia	0.0758
OTU126: Prevotella	0.0043
OTU143: Corynebacterium	0.2575
OTU169: Aggregatibacter	0.1500
OTU221: Enterobacteriaceae	0.0415
OTU233: Neisseria	0.0255
OTU240: Leptotrichia	0.0086
OTU264: Veillonella	0.0684
OTU272: Haemophilus	0.6663
OTU286: Neisseriaceae	0.1457
OTU295: Enterobacteriaceae	0.0126
OTU309: Neisseria	0.0070
OTU311: Enterobacteriaceae	0.0186
OTU316: Prevotella	0.0279
OTU323: Treponema	0.0144
OTU326: Enterobacteriaceae	1.4293
OTU339: Leptotrichia	0.0756
OTU341: Enterobacteriaceae	0.0177
OTU360: Capnocytophaga	0.0483
OTU363: Leptotrichia	0.1112
OTU379: Leptotrichia	0.1011
OTU388: Paludibacter	0.0413
