# Supplementary material for: Salivary microbial changes during the first 6 months of orthodontic treatment
Source: PeerJ. 2020 Dec 1;8:e10446. doi: 10.7717/peerj.10446 (PMC7718796; doi:10.7717/peerj.10446)
Supplement: Supplemental Information 6 [file peerj-08-10446-s006.pdf]

Table S2-2 Information of hub nodes (T1)

OTU ID and taxa	Relative abundance (%)
OTU003: Selenomonas	0.1574
OTU019: Prevotella	0.0980
OTU022: Mogibacteriaceae	0.0144
OTU028: Prevotella	0.0505
OTU029: Rs-045	0.0322
OTU030: Actinomyces	0.0187
OTU031: Treponema	0.0179
OTU050: Treponema	0.0331
OTU058: Schwartzia	0.0322
OTU101: Treponema	0.0085
OTU126: Prevotella	0.0097
OTU143: Corynebacterium	0.3470
OTU169: Aggregatibacter	0.1286
OTU240: Leptotrichia	0.0154
OTU251: Leptotrichia	0.1061
OTU259: Prevotella	0.0888
OTU275: Prevotella	0.3377
OTU279: Prevotella	0.1557
OTU287: Fusobacterium	0.0667
OTU316: Prevotella	0.0452
OTU323: Treponema	0.0157
OTU336: Prevotella	0.2215
OTU347: Weeksellaceae	0.0096
OTU358: Atopobium	0.0254
OTU363: Leptotrichia	0.2135
OTU379: Leptotrichia	0.1289
OTU390: TM7-3	0.0590
OTU401: Leptotrichia	0.1980
OTU405: Megashaera	0.0683
OTU408: Selenomonas	0.3054
