# Supplementary material for: Salivary microbial changes during the first 6 months of orthodontic treatment
Source: PeerJ. 2020 Dec 1;8:e10446. doi: 10.7717/peerj.10446 (PMC7718796; doi:10.7717/peerj.10446)
Supplement: Supplemental Information 7 [file peerj-08-10446-s007.pdf]

Table S2-3 Information of hub nodes (T2)

OTU ID and taxa	Relative abundance (%)
OTU018: Oribacterium	0.0996
OTU027: Moryella	0.0367
OTU048: Mogibacterium	0.0159
OTU096: Selenomonas	0.6662
OTU118: Prevotella	0.6066
OTU162: Lachnonaerobaculum	0.1908
OTU186: Megasphaera	0.5214
OTU194: Prevotella	0.3762
OTU201: Prevotella	0.1002
OTU209: Prevotella	0.1499
OTU214: Prevoella	0.6329
OTU239: Prevotella	0.2639
OTU250: Veillonella	0.2178
OTU257: Leptotrichia	0.2600
OTU259: Prevotella	0.0264
OTU260: Prevotella	0.1372
OTU271: Prevotella	0.0043
OTU284: Prevotella	0.0574
OTU293: Atopobium	0.2200
OTU321: Veillonella	5.0982
OTU325: Prevotella	0.0471
OTU329: Mogibacteriaceae	0.1118
OTU335: Prevotella	0.0235
OTU340: Bulleidia	0.1850
OTU343: Prevotella	0.0084
OTU368: Prevotella	7.5719
OTU374: Prevotella	0.0055
OTU376: Prevotella	0.0141
OTU392: Clostridiales	0.0850
